# Targeting Pin1 by All-Trans Retinoic Acid (ATRA) Overcomes Tamoxifen Resistance in Breast Cancer via Multifactorial Mechanisms

**DOI:** 10.3389/fcell.2019.00322

**Published:** 2019-12-06

**Authors:** Songyin Huang, Yang Chen, Zhi-Mei Liang, Na-Na Li, Yujie Liu, Yinghua Zhu, Dingzhun Liao, Xiao Zhen Zhou, Kun Ping Lu, Yandan Yao, Man-Li Luo

**Affiliations:** ^1^Department of Clinical Laboratory, Sun Yat-sen Memorial Hospital, Sun Yat-sen University, Guangzhou, China; ^2^Guangdong Provincial Key Laboratory of Malignant Tumor Epigenetics and Gene Regulation, Sun Yat-sen Memorial Hospital, Sun Yat-sen University, Guangzhou, China; ^3^Medical Research Center, Sun Yat-sen Memorial Hospital, Sun Yat-sen University, Guangzhou, China; ^4^Breast Tumor Center, Sun Yat-sen Memorial Hospital, Sun Yat-sen University, Guangzhou, China; ^5^Department of Pathology, Sun Yat-sen Memorial Hospital, Sun Yat-sen University, Guangzhou, China; ^6^Division of Translational Therapeutics, Department of Medicine, Beth Israel Deaconess Medical Center, Harvard Medical School, Boston, MA, United States

**Keywords:** ATRA, Pin1, breast cancer, tamoxifen, ERα

## Abstract

Breast cancer is the most prevalent tumor in women worldwide and about 70% patients are estrogen receptor positive. In these cancer patients, resistance to the anticancer estrogen receptor antagonist tamoxifen emerges to be a major clinical obstacle. Peptidyl-prolyl isomerase Pin1 is prominently overexpressed in breast cancer and involves in tamoxifen-resistance. Here, we explore the mechanism and effect of targeting Pin1 using its chemical inhibitor all-trans retinoic acid (ATRA) in the treatment of tamoxifen-resistant breast cancer. We found that Pin1 was up-regulated in tamoxifen-resistant human breast cancer cell lines and tumor tissues from relapsed patients. Pin1 overexpression increased the phosphorylation of ERα on S118 and stabilized ERα protein. ATRA treatment, resembling the effect of Pin1 knockdown, promoted ERα degradation in tamoxifen-resistant cells. Moreover, ATRA or Pin1 knockdown decreased the activation of ERK1/2 and AKT pathways. ATRA also reduced the nuclear expression and transcriptional activity of ERα. Importantly, ATRA inhibited cell viability and proliferation of tamoxifen-resistant human breast cancer cells *in vitro*. Slow-releasing ATRA tablets reduced the growth of tamoxifen-resistant human breast cancer xenografts *in vivo*. In conclusion, ATRA-induced Pin1 ablation inhibits tamoxifen-resistant breast cancer growth by suppressing multifactorial mechanisms of tamoxifen resistance simultaneously, which demonstrates an attractive strategy for treating aggressive and endocrine-resistant tumors.

## Introduction

Breast cancer is a leading cause of cancer-related death in female (Chen et al., 2016). In all breast cancer patients, approximately 70% patients are estrogen receptor positive (Deroo and Korach, 2006; Yager and Davidson, 2006; Nilsson et al., 2011). Although selective estrogen receptor modulator such as tamoxifen are effective for ER positive patient, approximately 30% of patients are not sensitive to tamoxifen treatment at the beginning, and over 50% of initial effective patients finally suffer from tamoxifen-resistance (TAMR) (Osborne and Schiff, 2011). The mechanism of TAMR is still not completely known. The possible molecular mechanisms include, but not limited to, the alteration of estrogen receptor transcriptional co-regulatory proteins (Shao et al., 2004; Girault et al., 2006), cross-talk between receptor tyrosine kinase signaling and estrogen receptor (Stenoien et al., 2001), non-canonical transcriptional activation of estrogen receptor (Anbalagan and Rowan, 2015), the expression of specific microRNAs (Miller et al., 2008), etc. Given that many studies have demonstrated that estrogen receptors play a central role in TAMR (Wijayaratne and McDonnell, 2001; Marsh et al., 2017; Zhang et al., 2017), blocking estrogen receptor related pathways is an attractive strategy to treat TAMR breast cancer.

Pin1 is a peptidyl-prolyl cis/trans isomerase (PPIase), which specifically recognizes pSer/Thr-Pro motifs of proteins and catalyzes their *trans*-*cis* conformational change (Lu and Zhou, 2007). Pin1 plays a vital role in cancer development by regulating more than 40 oncoproteins and over 20 tumor suppressors, therefore promoting cancer growth and cancer stem cell tumorigenesis (Zhou and Lu, 2016). Pin1 has been found to be up-regulated in tamoxifen-resistant breast cancer (Stanya et al., 2008; Namgoong et al., 2010; Khanal et al., 2012). Overexpression of Pin1 reduces the protein stability of estrogen receptor transcriptional co-regulatory protein SMRT (Stanya et al., 2008), as well as regulates the transcription function of ERα (Rajbhandari et al., 2012, 2015). Knockdown of Pin1 by siRNA inhibits the viability of TAMR breast cancer cells (Namgoong et al., 2010), indicating that Pin1 might be a promising therapeutic target for tamoxifen-resistant breast cancer. However, due to the lack of appropriate Pin1 inhibitors, it is challenging to evaluate the effect of targeting Pin1 on overcoming TAMR. Recently, Wei et al. has discovered all-trans retinoic acid (ATRA) as a specific Pin1 chemical inhibitor (Wei et al., 2015). ATRA has been used to induce differentiation and treat acute promyelocytic leukemia (APL). In APL, ATRA facilitates PML–RAR-α degradation, thereby suppresses APL stem cells (Huang et al., 1988; de The and Chen, 2010; Sanz and Lo-Coco, 2011). Wei et al. (2015) has found that besides RAR, Pin1 is a key target of ATRA in APL and breast cancer. ATRA directly and selectively binds to and degrades active Pin1, thereby inhibiting multiple Pin1-regulated cancer driving pathways.

In the current study, we explored the effects of ATRA in inhibiting Pin1 and treating tamoxifen-resistant breast cancer *in vitro* and *in vivo*. Our experiments showed that Pin1 was up-regulated in tamoxifen-resistant cells and increased ERα protein stability. ATRA treatment accelerated ERα protein turnover, reduced ERα transcriptional activity, and decreased the phosphorylation of AKT and ERK1/2 simultaneously, which further inhibited ERα activation. Thus, ATRA induced the degradation of Pin1 and suppressed cell viability and proliferation of tamoxifen-resistant breast cancer cells. More importantly, slow-releasing ATRA tablets showed remarkable anti-tumor effects in the tamoxifen-resistant xenograft model. Therefore, targeting Pin1 by ATRA promised a new potential approach to treat tamoxifen-resistant breast cancer.

## Materials and Methods

### Cell Culture

The human breast cancer cell lines MCF7 and T47D were purchased from American Type Culture Collection (Manassas, VA, United States) and cultured in DMEM (Thermo Fisher Scientific, Waltham, MA, United States) supplemented with 10% fetal bovine serum (FBS, GBICO). Tamoxifen resistant cell lines (MCF-7R and T47DR) were kindly provided by Dr. Qiang Liu as gift, and were cultured in no-phenol red 1640 medium (Life Technologies, United States) supplemented with 10% charcoal-stripped FBS (cFBS) (HyClone, United States) and 1 μM 4-hydroxytamoxifen (Sigma-Aldrich, St. Louis, MO, United States). Cells were maintained at 37°C in a humidified atmosphere containing 5% CO_2_.

### Antibodies, Reagents, and Sequences

Antibodies of ERα was from cell signaling technology (8644) and Abcam (16660). Antibodies of Pin1 was from R&D (MAB2294) and Abnova (MAB12340). Phospho-ERα S118 antibody was from Abcam (ab32396). Phospho-ERα S167 antibody was from cell signaling technology (64508). ATRA was from Sigma (R2625). Tamoxifen was from Sigma (H6278). Pin1 shRNA targeting sequence: CCACCGTCACACAGTATTTAT; Pin1 siRNA-1 targeting sequence: TCAGGCCGAGTGTACTACT; Pin1 siRNA-2 targeting sequence: GCTCAGGCCGAGTGTACTA; RARα siRNA-1 targeting sequence: CCAGCTCACAGAACTGCTT; RARα siRNA-2 targeting sequence: TTCCGCACGTAGACCTT TAGC; ERα siRNA-1 targeting sequence: CAGGCCAAATTCA GATAAT; ERα siRNA-2 targeting sequence: GGTCCAC CTTCTAGAATGT.

### Colony-Forming Assays

Six-well plates were seeded 2000 cells per well. Cells were treated with vehicle (DMSO), 10 μM ATRA, 10 μM tamoxifen (TAM) or ATRA plus TAM, and medium were changed every 3 days. 14 days later cells were fixed with methanol, stained with 0.5% crystal violet.

### Cell Viability Assays

Cell viability was measured using the Cell Titer Glo reagent (Promega). The cells were plated in 96-well plates at 1 × 10^3^ cells per well and maintained at 37°C. At the indicated time points, cell viability was measured according to the manufacturer’s instructions.

### Quantitative RT-PCR

Total RNA was extracted from cells using Trizol (life, United States). MMLV kit (life, United States) was used to generate cDNA. Real time PCR were performed using Toyobo SYBR GREEN. The primers used were as follows: Pin1 (forward, 5′- AGCTCAGGCCGAGTGTACTA-3′; reverse, 5′-CCTTGGTCCGGGTGATCTTC -3′); growth regulation by estrogen in breast cancer 1 (GREB1) (forward, 5′-GTGGT AGCCGAGTGGACAAT-3′; reverse, 5′-ATTTG TTTCCAGCC CTCCTT-3′); progesterone receptor (PGR) (forward, 5′-GG CATGGTCCTTGGAGGT -3′; reverse, 5′-CCACTGGCTGTGG GAGAG-3′); c-Myc (forward, 5′- TACAACACCC GAGC AAGGAC-3′; reverse, 5′-GAGGCTGCTGGTTTTCCACT-3′); β-actin (forward, 5′-GGAAGGGGGACGGGGACAGC-3′; reverse, 5′- GGAGGAGCAAG GAGCGGGAG-3′).

### Immunoblot Analysis

Cells were lysed with RIPA buffer containing 0.1% protease inhibitors or phosphatase inhibitors (Life, United States). The supernatant of lysate was separated by electrophoresis and blotted onto a PVDF membrane, then blocked with 5% skim milk at room temperature for 1 h. The blots were incubated with the following antibody at 4°C overnight: ERα (1:1000, CST, #8644); Pin1 (1:1000, R&D, #MAB2294); phospho-ERα S118 (1:1000, CST, #ab32396); AKT (1:1000, CST); Flag-tag (1:5000, Sigma, United States); phospho-AKT (1:1000, CST); phospho-c-Raf (1:1000, CST); phospho-MEK1/2 (1:1000, CST); ERK1/2 (1:1000, CST); phospho-ERK1/2 (1:1000, CST); phospho-ERαS167 (1:1000, CST, #64508); β-Actin (1:2000, CST); GAPDH (1:2000, proteintech). After incubation with HRP-conjugated secondary antibodies at room temperature for 1 h, all blots were detected by an enhanced chemiluminescence (ECL) and were scanned using ChemiDoc^TM^ XRS + imaging system (Bio-Rad, Hercules, CA, United States).

### Immunofluorescence

MCF-7R and T47DR cells were fixed in 4% polyoxymethylene at 4°C for 20 min, washed with PBS and permeabilized in 0.1% Triton X-100 at room temperature for 10 min. Cells were then blocked in 10% goat serum at room temperature for 30 min, and incubated with ERα antibody (1:100, Abcam, #16660) in 10% goat serum at 4°C overnight. Cells were washed, incubated with secondary antibodies at room temperature for 1 h, washed, incubated with DAPI at room temperature for 15 min. Slides were then covered with fluorescently quencher 30 μl, sealed and photographed with an Olympus confocal microscope.

### Animal Experiments

Nude mice were purchased from Laboratory Animal Service Center, Sun Yat-sen University. The experiment protocol was approved by the Animal Care and Use Committee of Sun Yat-sen University. 2 × 10^6^ MCF-7R cells were mixed with an equal volume of matrigel (Corning) and injected into the mammary fat pads of 4 week-old female BALB/c nude mice. One week later, when tumor size reached ∼100 mm^3^, the tumor-bearing mice were randomized into treatment groups. 21-days ATRA tablets were implanted under neck skin. Tamoxifen was injected at 4 mg/kg per day. Tumor volume was measured every 3 days.

### Patients and Immunohistochemistry

Tumor samples were obtained from patients with ER positive breast cancer who underwent tamoxifen therapy in Sun Yat-sen Memorial Hospital. All samples were collected from patients with informed consent, and all related procedures were performed with the approval of the internal review and ethics boards of Sun Yat-sen Memorial Hospital. Immunohistochemistry staining for Pin1 and ERα was performed as described previously (Luo et al., 2015; Zhang et al., 2016). Briefly, sodium citrate was used to repair tissue antigen. Incubation of primary antibodies (Pin1, 1:50, Abnova, #MAB12340; ERα, 1:50, CST, #16660) was carried out at 4°C overnight. The slides were incubated with HRP-conjugated secondary antibodies at room temperature for 1 h, washed, visualized with DAB solution, followed by staining with hematoxylin. Immunostaining results was analyzed by ImageJ software.

### Statistical Analyses

All data are presented as the means ± SD. Student’s *t*-test was used to analysis the significance between two experimental groups, and ANOVA test was used to analysis among three or more groups. *P* < 0.05 was considered significant. All the statistical analyses were performed using SPSS20.

## Results

### Pin1 Is Up-Regulated in Tamoxifen-Resistant Breast Cancer and Correlates With ERα Expression in Human Breast Cancer Cell Lines and Cancer Tissues

**FIGURE 1:**
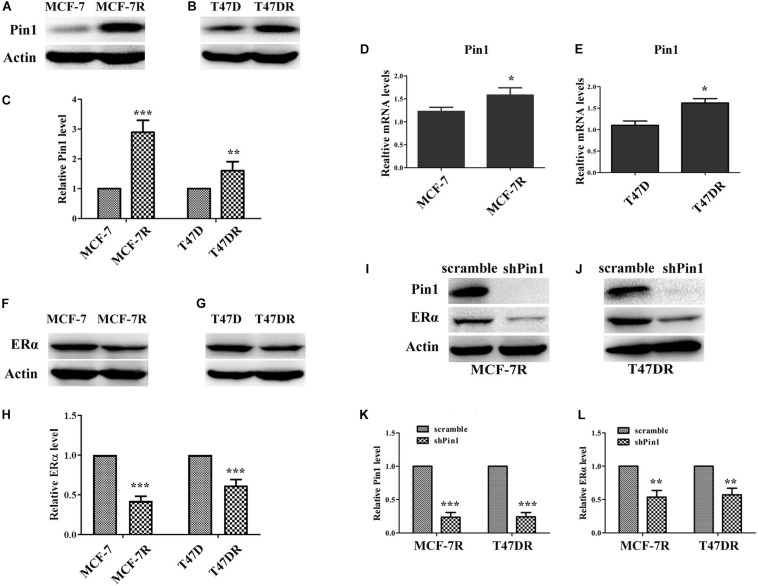
Pin1 is overexpressed in tamoxifen resistant breast cancer cells. **(A,B)** Pin1 is up-regulated in tamoxifen resistant breast cancer cells. Pin1 protein was detected by western blot in parental (MCF-7 and T47D) and tamoxifen resistant (MCF-7R and T47DR) cells. **(C)** Quantification of Pin1 levels in parental and tamoxifen resistant cells. Western blot bands in panels **(A,B)** were quantified by densitometric scan and represented as a relative ratio to control samples. Data are represented as means ± SD for three independent experiments. **(D,E)** Pin1 mRNA is up-regulated in tamoxifen resistant breast cancer cells, as detected by qRT-PCR. **(F–H)** The ERα protein level in parental and resistant breast cancer cells. Western blot bands were quantified in panel **(H)**. **(I–L)** Pin1 knockdown decreases the level of ERα in MCF-7R and T47DR cells. Western blot bands were quantified in panels **(K,I)**. ^∗^*P* < 0.05, ^∗∗^*P* < 0.01, ^∗∗∗^*P* < 0.001.

We established tamoxifen-resistant human breast cancer cell lines MCF-7 and T47D by long-term exposure to tamoxifen (Herman and Katzenellenbogen, 1996; Knowlden et al., 2003; Chu et al., 2015). We confirmed the resistance of these cells by showing that the viability of resistance cells was significantly higher than parental cells and apoptosis were remarkable lower in the presence of 1 μM tamoxifen (Chu et al., 2015). We found that both Pin1 protein and mRNA were up-regulated in tamoxifen-resistant MCF-7 (MCF-7R) and T47D (T47DR) cells, comparing to parental cells (Figures 1A–E and Supplementary Figure S5), which was consistent with previous reports that Pin1 was overexpressed in TAMR human breast cancer tissues (Namgoong et al., 2010; Khanal et al., 2012).

Although ERα was not so indispensable for TAMR cells as for parental cells, depleting ERα still further limited the growth of TAMR cells (Xiong et al., 2017). Indeed, through a variety of mechanisms, TMAR breast cancer cells made full use of remaining ERα to escape from the impact of tamoxifen (Osborne and Schiff, 2005; Johnston, 2010; Marsh et al., 2017). Here we examined the ERα level in TAMR cells, and found that ERα protein was down-regulated in TAMR cells (Figures 1F–H and Supplementary Figure S5), as shown previously (Stone et al., 2013; Lu et al., 2016). Given that ERα was a known Pin1 substrate which was positively regulated by Pin1 (Rajbhandari et al., 2012, 2015). We asked why Pin1 level was high while ERα level was low in TAMR cells. We found that Pin1 knockdown further decreased ERα level in TAMR cell lines (Figures 1I–L and Supplementary Figure S5). These results suggest that Pin1 is up-regulated and helps maintain ERα levels in TAMR cells even although ERα levels in these cells are low.

**FIGURE 2:**
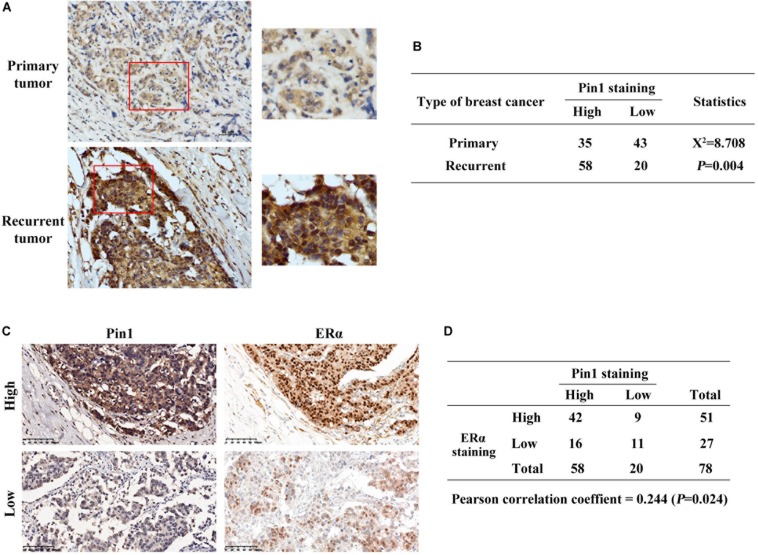
Pin1 is up-regulated in recurrent tumors and positively correlates with ERα in breast cancer tissues. **(A)** Representative immunohistochemistry image shows that Pin1 expression is higher in recurrent tamoxifen treated tumors than primary breast cancer tissues. Tissues in the red frames in left panels were magnified in the right panels. Scale bars, 50 μm. **(B)** Statistic analysis shows that Pin1 expression is significantly different in primary and recurrent tumors. *P* = 0.004. **(C,D)** Pin1 expression correlates with ERα expression in recurrent breast cancer tissues. Representative image **(A)** showed high and low levels of Pin1 and ERα. Pearson correlation of Pin1 and ERα was analyzed in panel **(B)**. Scale bars, 100 μm.

Next, we detected Pin1 and ERα protein levels in tumor tissues of recurrent ER positive breast cancer patients, who have received tamoxifen treatment. Pin1 protein level was significantly higher in recurrent tumors comparing with primary tumors (*P* = 0.004) (Figures 2A,B). More importantly, the expression level of Pin1 was associated with ERα in these tissues (Figures 2C,D). Together, Pin1 was up-regulated in both TAMR human breast cancer cell lines and relapsed tumor tissues, which positively correlated with ERα expression.

### ATRA-Induced Pin1 Ablation Promotes ERα Protein Degradation

**FIGURE 3:**
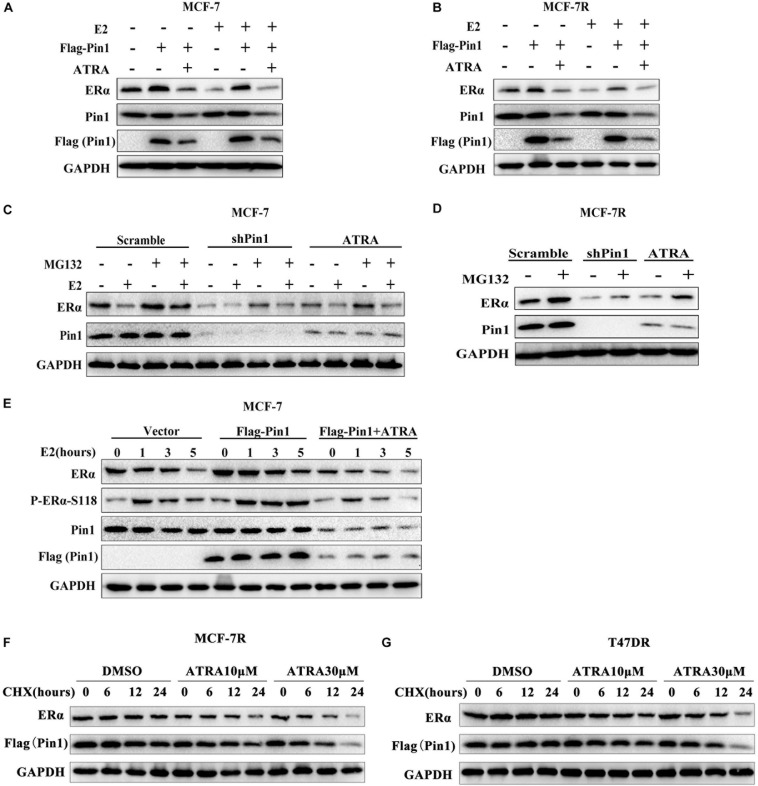
ATRA promotes proteasome-mediated degradation of ERα by blocking Pin1. **(A,B)** Ectopic expression of Pin1 up-regulates ERα, but ATRA abrogates the effect. MCF-7 **(A)** and MCF-7R **(B)** cells transfected with Flag-Pin1 or empty vector were pretreated with 10 μM ATRA for 72 h, followed by 10nM E2 or EtOH treatment for 5 h. **(C,D)** Pin1 knockdown or ATRA treatment promotes ERα degradation. MCF-7 **(C)** or MCF-7R **(D)** were treated with 10 μM MG132 for 4 h before harvesting. **(E)** Overexpression of Pin1 stabilizes pS118-ERα, but ATRA abrogates the effect. MCF-7 cells transfected with Flag-Pin1 or empty vector were pretreated with 10 μM ATRA for 72 h, followed by 10nM E2 treatment for 0, 1, 3, and 5 h. **(F,G)** ATRA promotes Pin1 and ERα degradation in MCF-7R and T47DR cells. Cells were pretreated with ATRA and treated with CHX for indicated time course.

To explore the effect of Pin1 on regulating ERα, and more importantly to test whether ATRA was effective in inhibiting Pin1’s function on ERα, we first examined whether overexpressing Pin1 affected ERα protein level. As expected, estradiol (E2) could induce down-regulation of ERα protein (Figures 3A,B and Supplementary Figure S6), which was due to ligand-dependent degradation (Wijayaratne and McDonnell, 2001; Nonclercq et al., 2004). We found that not only enforced Pin1 expression (Flag-Pin1) rescued the ERα expression, but the Pin1 inhibitor ATRA reversed this effect in both MCF-7 and MCF-7R cells (Figures 3A,B and Supplementary Figures S1A,B), suggesting that ATRA specifically inhibiting Pin1 from protecting the degradation of ERα.

Next, to confirm the effect of ATRA on ERα protein degradation, MCF-7 and MCF-7R cells stably knocking down Pin1 with shRNA were treated with or without proteasome inhibitor MG132. Contrary to overexpression experiments, Pin1 knockdown promoted E2-induced ERα degradation (Figures 3C,D and Supplementary Figures S1C,D). Notably, ATRA had the same effects as shPin1 both in MCF-7 and MCF-7R cells (Figures 3C,D and Supplementary Figures S1C,D). One of the vital regulatory element governing ERα protein turnover is Ser118 phosphorylation of the N-terminus, which is phosphorylated by ERK1/2 as well as other kinases, and regulated by Pin1 (Rajbhandari et al., 2012, 2014, 2015). Therefore we speculated that ATRA promoted ERα protein turnover through ERα-pS118. Indeed, overexpressing Pin1 increased the phosphorylation of S118 as well as total ERα level, suggesting that Pin1 prevented the turnover of pERα, whereas ATRA reversed Pin1’s effect (Figure 3E and Supplementary Figures S1E,F).

To directly examine whether ATRA could promote the degradation of Pin1 and ERα in tamoxifen-resistant cells, we treated MCF-7R and T47DR cells with ATRA, followed by cycloheximide (CHX) and detected the protein levels at different time points. Our data showed that ATRA promoted the degradation of both Pin1 and ERα in TAMR breast cancer cells in a dose dependent manner (Figures 3F,G and Supplementary Figures S1G–J). In addition, we treated MCF-7R and T47DR cells with increasing doses of ATRA for different length of time, and found that both Pin1 and ERα protein levels indeed reduced (Supplementary Figures S2A,B, S9). Together, these data demonstrate that overexpressing Pin1 in breast cancer cells protects the ERα protein from degradation. ATRA blocks the up-regulated Pin1 in tamoxifen-resistant cells, thereby promoting the degradation of remaining ERα in tamoxifen-resistant cells, which suggests that ATRA may be able to overcome TAMR by eradicate ERα.

### ATRA Blocks ERK1/2 and AKT Pathways in TAMR Breast Cancer Cells

**FIGURE 4:**
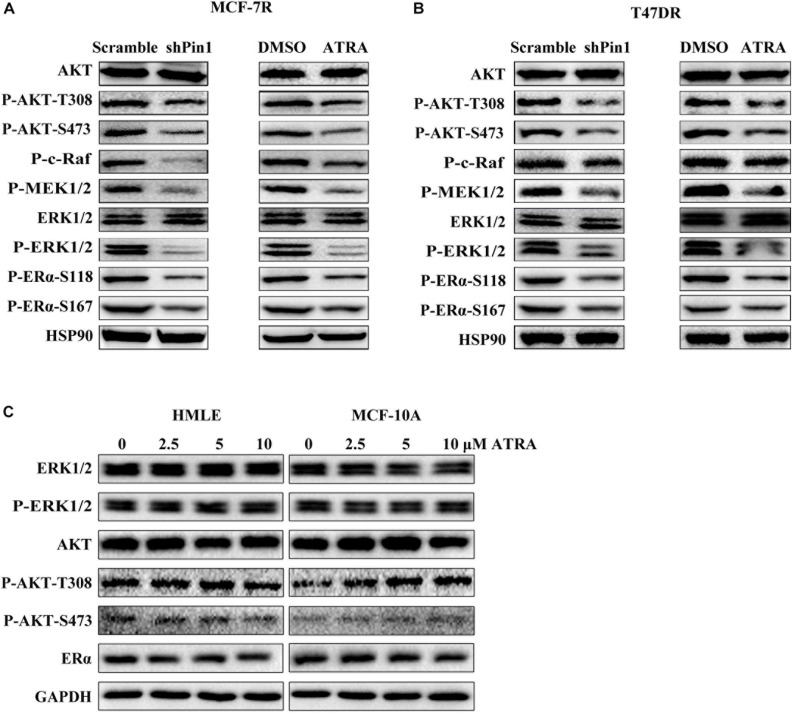
ATRA and Pin1 knockdown inhibits the activation of AKT and ERK1/2 signal pathways. **(A)** Pin1 knockdown or ATRA treatment inhibits the activation of AKT and ERK1/2 signal pathways simultaneously in MCF-7R cells. Cells were infected with lentivirus expressing scramble or Pin1 shRNA, or treated with 10 μM ATRA for 72 h. **(B)** Pin1 knockdown or ATRA treatment decreases the activation of AKT and ERK1/2 signal pathways simultaneously in T47DR cells. **(C)** ATRA treatment doesn’t affect ERα, AKT, and ERK1/2 in HMLE and MCF-10A cells.

Several kinase pathways have been reported to involve in the growth of TAMR breast cancer cells, including AKT and ERK1/2 (Svensson et al., 2005; Garcia-Becerra et al., 2012). AKT phosphorylates ERα on S167 (Sun et al., 2001), and ERK1/2 phosphorylates ERα on S104/S106, S167, and S118 (Ali et al., 1993; Arnold et al., 1995; Endoh et al., 1999; Sun et al., 2001; Sheeler et al., 2003). Notably, phosphorylation of S118 and S167 induces estrogen-independent activation of ERα (Garcia-Becerra et al., 2012). Moreover, AKT and ERK1/2 activity are also regulated by Pin1 (Liao et al., 2009; Luo et al., 2015). Thus, we explored the effects of ATRA in inhibiting these pathways in MCF-7R and T47DR cells. ATRA treatment didn’t alter the total expression of AKT or ERK1/2, but reduced the level of phosphorylated AKT, MEK1/2, ERK1/2 and Raf (Figures 4A,B). In consistence with decreased activity of these pathways, phosphorylation of ERα on S167 and S118 were also inhibited, resembling the effect of Pin1 knockdown (Figures 4A,B and Supplementary Figure S7).

To assess the effect of ATRA on ER signaling in normal cells, we treated immortalized mammary epithelial cells MCF-10A and HMLE with different doses of ATRA. These two cell lines expressed very low level of Pin1, comparing to breast cancer cell lines (Wei et al., 2015). We found that ATRA almost had no effect on the protein level of ERα, or P-ERK1/2 and P-AKT (Figure 4C). Hence, ATRA had the unique potential to simultaneously block multiple signal pathways in TAMR breast cancer cells.

In addition, RARα, another ATRA target, has been indicated to play a role in tamoxifen resistance of breast cancer (Johansson et al., 2013). Using siRNAs, we knocked down either Pin1 or RARα in MCF-7R cells with or without ATRA treatment (Supplementary Figures S3A,B). The total and phosphorylated levels of ERα only decreased in Pin1-silencing, but not RARα-silencing cells (Supplementary Figures S3C, S10). As ATRA can still target other proteins to regulate ERα, our data indicate that ATRA may mainly act on Pin1 to regulate ERα.

### ATRA Inhibits ERα Transcriptional Activity

**FIGURE 5:**
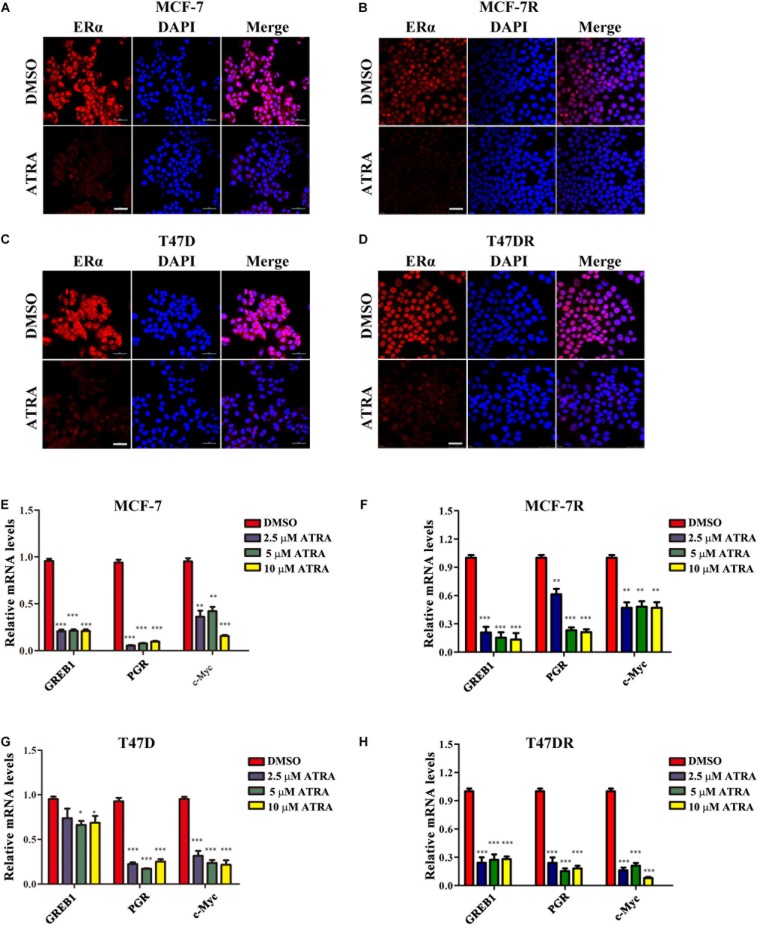
ATRA suppresses nuclear expression and transactivation of ERα. **(A,B)** ATRA decreases the level of nuclear ERα in MCF-7 and MCF-7R. Cells were treated with 10 μM ATRA for 72 h before immunofluorescence staining. Scale bars, 50 μm. **(C,D)** ATRA decreases the level of nuclear ERα in T47D and T47DR. Cells were treated with10 μM ATRA for 72 h before immunofluorescence staining. Scale bars, 50 μm. **(E–H)** ATRA suppresses the transcription of ERα target genes GREB1, PGR, and c-Myc. Cells were treated with ATRA (2.5, 5.0, and10 μM) for 48 h. Expression of ERα downstream genes were detected by qRT-PCR, and normalized to β-ACTIN expression in DMSO treated cells. Error bars denote the SD of three biological replicates, ^∗^*P* < 0.05, ^∗∗^*P* < 0.01, ^∗∗∗^*P* < 0.001.

To determine whether ATRA affected the transcriptional function of ERα in tamoxifen-resistant cells, we first examined ERα subcellular expression by immunofluorescence. Parental or resistant MCF-7 and T47D cells were treated with 10 μM ATRA for 72 h. The nuclear staining of ERα was dramatically reduced by ATRA treatment in all cell lines (Figures 5A–D), indicating a decreased transcriptional activity of ERα. Next, we detected the transcription of three known ERα regulatory genes, including PGR, GREB1, and c-Myc (Lee and Gorski, 1996; Bosch et al., 2015; Wu et al., 2018). The mRNA levels of these three genes were decreased after ATRA treatment (Figures 5E–H). These data suggest that ATRA suppresses ERα transcriptional function *in vitro*.

### ATRA Inhibits the Viability and Proliferation of Parental and Tamoxifen-Resistant Breast Cancer Cells

**FIGURE 6:**
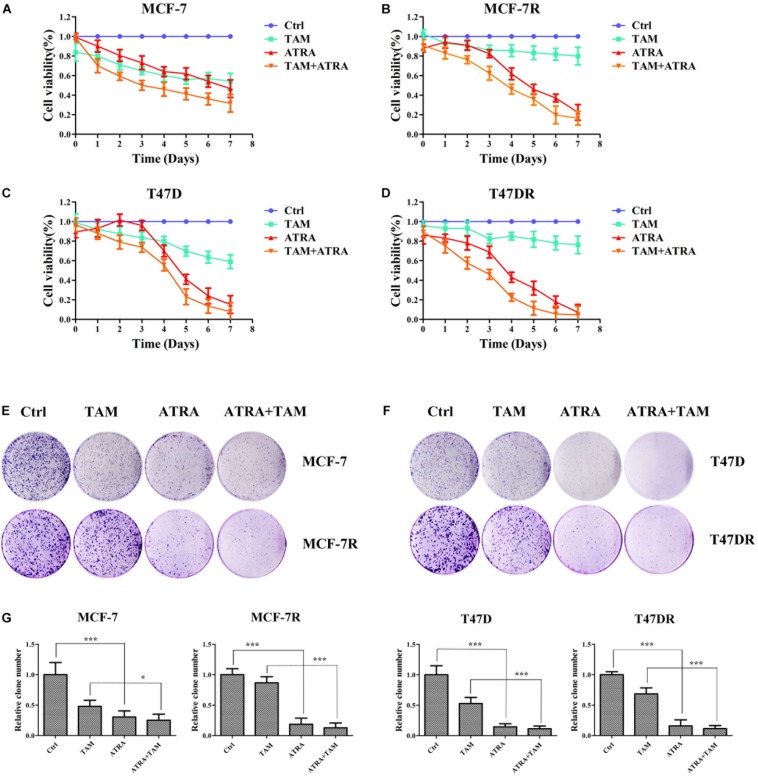
ATRA inhibits the cell growth of tamoxifen resistant breast cancer cells. **(A–D)** The inhibitory effect of ATRA and tamoxifen on cell viability of parental and tamoxifen resistant breast cancer cells. Viability of drug treated cells was normalized to control untreated cells (Ctrl). **(E,F)** The inhibitory effect of ATRA and tamoxifen on foci formation of parental and tamoxifen resistant breast cancer cells. **(G)** Quantification of foci formation by ImageJ software. ^∗^*P* < 0.05 and ^∗∗∗^*P* < 0.001, as determined by Student’s *t*-test. Bar graphs are means ± SD by three independent experiments.

Although our data demonstrated that ATRA targeted Pin1 to promote ERα protein degradation, decrease ERα transcriptional activity, and inhibit AKT and ERK1/2 pathway, the therapeutic potential of ATRA in treating tamoxifen-resistant breast cancer was still not clear. We thus evaluated the effects of ATRA on cell viability and foci formation of parental and TAMR cells. As expected, tamoxifen treatment reduced the growth of parental cells, but not the TAMR cells, whereas ATRA suppressed the proliferation of both parental and TAMR cells (Figures 6A–D). Moreover, ATRA potentiated tamoxifen therapeutic effect in both parental and TAMR cells (Figures 6A–D). In the colony formation experiments, ATRA showed similar effects as in the proliferation assay (Figures 6E–G).

We have shown that ATRA-induced Pin1 degradation reduces the protein expression of ERα in tamoxifen-resistant breast cancer cells. To confirm that ERα contributes to tamoxifen resistance in our TAMR cell model, we used siRNAs to knock down ERα in MCF-7R and T47DR. ERα siRNAs dramatically suppressed the proliferation of these cells upon tamoxifen treatment (Supplementary Figures S4A,B, S10), suggesting that ERα indeed contributed to TAMR in these cells.

**FIGURE 7:**
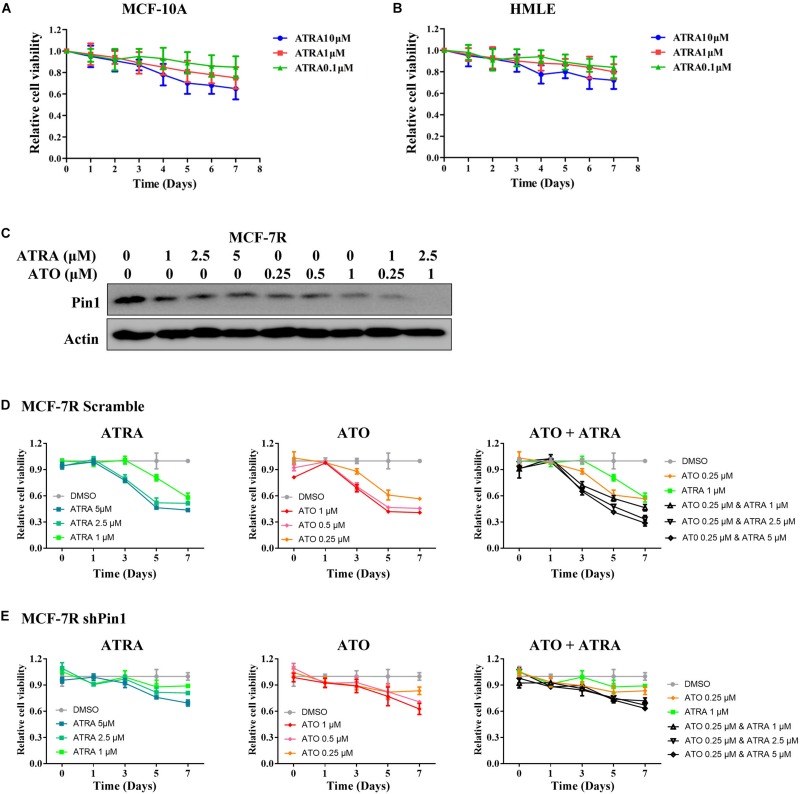
ATRA and other Pin1 inhibitor has limited inhibitory effect on Pin1-low expressing cells. **(A,B)** ATRA shows little inhibitory effect on the viability of MCF-10A and HMLE cells. Cells were treated with increasing doses of ATRA for 7 days. **(C)** The effect of ATRA and ATO on reducing the level of Pin1 protein in MCF-7R cells. The concentration of ATRA and ATO were added to the culture medium as indicated for 48 h. **(D,E)** ATRA and ATO show much less inhibitory effect on the proliferation of shPin1 cells than that of control MCF-7R cells. MCF-7R cells stably expressing scramble or Pin1 shRNA were treated with ATRA and ATO for 7 days.

To investigate the effects of ATRA on Pin1-low cells, we treated MCF-10A and HMLE with different doses of ATRA. ATRA exhibited very limited inhibitory effects on cell viability of these epithelial cells (Figures 7A,B), likely because ATRA selectively targets active Pin1 in cancer cells, but not in normal cells with low Pin1 levels (Wei et al., 2015). Thus these results demonstrated that ATRA inhibits cell growth of TAMR breast cancer, with little effects on normal cells.

We also treated MCF-7R and T47DR cells that had knocked down Pin1 with ATRA and ATO, a newly identified Pin1 inhibitor (Kozono et al., 2018). Both ATRA and ATO showed much less inhibitory effect on the proliferation of shPin1 cells than that of control MCF-7R cells (Figures 7C–E and Supplementary Figure S8). Notably, although either ATRA or ATO could inhibit the proliferation of TAMR cells, the combination of ATRA + ATO effectively suppressed the cell viability in low dose (Figures 7D,E).

### ATRA Suppresses the Growth of TAMR Breast Cancer *in vivo*

**FIGURE 8:**
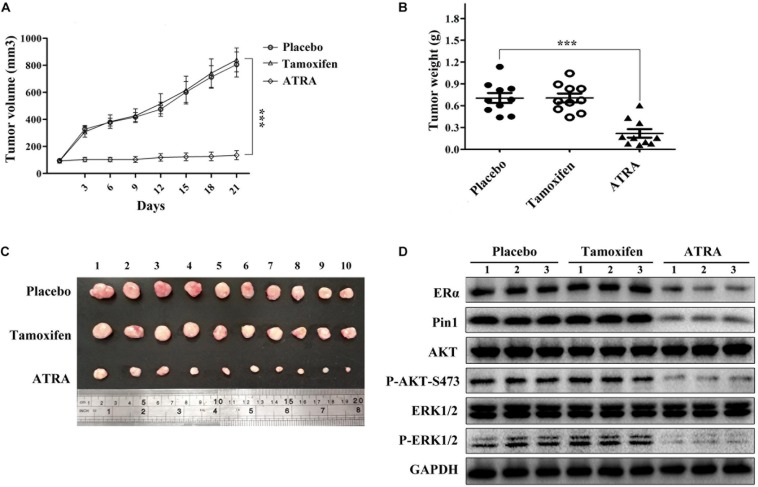
ATRA suppresses the growth of TAMR breast cancer *in vivo*. **(A)** Growth curve of MCF-7R xenografts in nude mice. Tumor volumes were measured every 3 days. Error bars represent SD of ten mice, ^∗∗∗^*P* < 0.001. **(B)** Slow-releasing ATRA pellet inhibits the tumor growth of MCF-7R xenografts in nude mice. The tumor weight represented the mean ± SD of each group, ^∗∗∗^*P* < 0.001. **(C)** MCF-7R xenografts were collected 3 weeks after treatment. **(D)** Representative immunoblots show Pin1, ERα, P-AKT, and P-ERK1/2 levels in MCF-7R xenografts.

Given the remarkable effects of ATRA on inhibiting ERα, AKT, and ERK1/2, as well as cell proliferation in tamoxifen-resistant breast cancer *in vitro*, we next asked whether ATRA had therapeutic effect against TAMR breast tumors *in vivo*. We established MCF-7R xenografts and implanted 21-day slow-releasing ATRA tablets in nude mice. Tamoxifen showed no therapeutic effect on TAMR xenografts, whereas ATRA remarkably inhibited the growth of TAMR breast cancer cells *in vivo* (Figures 8A–C). In addition, ATRA significantly suppressed Pin1, ERα, as well as the phosphorylation of AKT and ERK1/2 in the xenografts (Figure 8D and Supplementary Figure S8). Therefore, ATRA is effective in overcoming tamoxifen resistance *in vivo*.

## Discussion

Tamoxifen resistance is one of the major hurdles in treating breast cancer. A large body of evidence suggests that modulation of ERα pathway and activation of pro-survival pathways are important factors of tamoxifen resistance (Cui et al., 2015; Ferraiuolo et al., 2017; Marsh et al., 2017; Zhang et al., 2017). Our study demonstrated that Pin1 was up-regulated in tamoxifen-resistant breast cancer cells and relapsed breast cancer tissues. ATRA-induced Pin1 degradation decreased the protein stability and transcription activity of ERα, as well as reduced the phosphorylation of pro-survival kinases AKT and ERK1/2 in tamoxifen-resistant breast cancer cells. Moreover, targeting Pin1 by ATRA inhibited cell growth *in vitro*, and exhibited anti-tumor effects *in vivo* against tamoxifen-resistant breast cancer. Our data suggest that ATRA is a potent drug in treating tamoxifen-resistant breast cancer via suppressing multifactorial mechanisms of tamoxifen resistance.

Compelling evidence has demonstrated that decreased ERα expression and function contributes to intrinsic and acquired tamoxifen resistance (Cui et al., 2015; Ferraiuolo et al., 2017; Marsh et al., 2017; Zhang et al., 2017). Various clinical and experimental models suggest that tumor cells with acquired resistance to tamoxifen express low level of ERα (Stone et al., 2013; Lu et al., 2016). In the presence of tamoxifen, the resistant cells can still activate ERα, but through a ligand-independent way (He et al., 2018), or rely on non-ERα growth-promoting pathways for survival (Hur et al., 2004; Cannings et al., 2007; Mohseni et al., 2014). Thus the low level of ERα is one of the key resources of growth signal that are available for the resistant cells to utilize. Previous study showed that Pin1 inhibited phosphorylation-dependent ubiquitination and degradation of ERα in breast cancer cells (Rajbhandari et al., 2014). Here we found that Pin1 was up-regulated in tamoxifen-resistant breast cancer cells. This up-regulated Pin1 prevented ERα from degradation, which substantially enhanced the ERα level in tamoxifen-resistant cells. Although ERα level was low in these resistant cells, it would be even lower if Pin1 was not up-regulated. In our clinical samples, ERα expression was high in more than 60% of recurrent breast cancer tissues. This may be because Pin1 is frequently highly expressed in recurrent tumors, therefore preventing ERα from degradation, which substantially enhances the ERα level in relapsed tumors. Notably, this increased ERα, just as the low level of ERα in the resistant cells, is very likely activated via ligand independent way. This is supported by the evidence that phosphorylation of key serine residues of ERα, in particular serine 118 and 167, promotes re-activation of ERα in a ligand-independent manner (Garcia-Becerra et al., 2012). Pin1 has been reported to bind specifically to pS118 ERα to isomerize the serine118-proline119 bond (Rajbhandari et al., 2012). Therefore, Pin1 overexpression promotes the growth of tamoxifen-resistant breast cancer cells by up-regulating the ligand-independent ERα activity.

In addition to the effects on ERα stabilization, isomerization of phosphorylated ERα by Pin1 directly increases endogenous ERα DNA binding activity (Rajbhandari et al., 2015). Our study showed that inhibiting Pin1 by ATRA suppressed nuclear ERα expression and the transcription of ERα target genes. Moreover, previous data suggest that besides affecting ERα, Pin1 may promote tamoxifen resistance of breast cancer by activating growth-promoting pathways (Khanal et al., 2012), down-regulating SMRT (Stanya et al., 2008) and cyclin dependent kinase (Khanal et al., 2012), facilitating tumor angiogenesis (Kim et al., 2009b, 2012) and epithelial–mesenchymal transition (Kim et al., 2009a). However, few studies assess the potential of Pin1 inhibitor in treating TAMR breast cancer *in vitro* and *in vivo*. Our study showed that ATRA decreased ERα level both *in vitro* and *in vivo*. Moreover, ATRA down-regulated phosphorylation of ERα at S118 and two important pro-survival kinases ERK1/2 and AKT. Thus, ATRA inhibits Pin1 to overcome tamoxifen resistance in breast cancer cells at least at three levels: (1) promotes the degradation of ligand independent ERα, (2) suppresses the transactivation of ERα, (3) inhibits alternative growth pathways. Indeed, our results showed that ATRA exhibited potent anti-tumor activity against tamoxifen-resistant breast cancer *in vitro* and *in vivo*.

All-Trans Retinoic Acid has been used to treat APL for a long period of time. Recently Wei et al. (2015) has discovered that ATRA is a Pin1 inhibitor which binds to Pin1’s active site and accelerated its degradation. These findings make it possible to expand the application of ATRA to treat more types of cancer, especially solid tumors, because Pin1 is overexpressed in a wide range of human cancers and regulates multiple cancer-driving pathways (Lu and Hunter, 2014; Zhou and Lu, 2016). Besides Wei et al. demonstrated that ATRA-induced Pin1 ablation inhibits triple-negative breast cancer cell growth (Wei et al., 2015), Liao et al. also reported the anti-tumor effect of ATRA in hepatocellular carcinoma (HCC) *in vitro* and *in vivo* (Liao et al., 2017; Yang et al., 2018). New Pin1 inhibitors have also been discovered to suppress the growth of cancer cells (Campaner et al., 2017; Kozono et al., 2018), even the tumor-initiating cells, as Pin1 promotes the self-renewal of these stem-like cancer cells (Luo et al., 2014; Rustighi et al., 2014, 2017). Our data showed that ATRA suppressed the cell proliferation of tamoxifen-resistant breast cancer cells, and effectively reduced the tumor growth of tamoxifen-resistant xenografts by promoting ERα degradation, decreasing ERα transactivation, and inhibits the activation of ERK1/2 and AKT. Given that multiple survival pathways and factors contribute to tamoxifen resistance, blocking a single pathway may be ineffective to overcome the resistance. Therefore, ATRA may have the advantage of suppressing multifactorial mechanisms of tamoxifen resistance simultaneously.

Currently there are still obstacles of using ATRA to treat solid tumors in human. Regular ATRA has a half-life of only 45 min in humans. Although slow-releasing pellets can be implanted subcutaneously in mice, the formulation of ATRA pellet is different from that used for oral administration or intravenous injection in human therapies, and can’t be applied to human yet. Novel controlled releasing formulation of ATRA for effective cancer therapy are being developed actively (Westervelt et al., 2002; Tsimberidou et al., 2006; Yang et al., 2018). In addition, ATRA concentration is high in treating solid tumors. Similar to the previous report that the combination of ATRA + ATO inhibited tumor-initiating cells (Kozono et al., 2018), we found that this combination could reduce ATRA concentration and effectively inhibited the growth of TAMR cells. Thus, studies are ongoing to increase the efficacy of ATRA by improving its formulation, or using ATRA as part of combination therapies.

In summary, our data have shown for the first time that targeting Pin1 by ATRA effectively inhibits the growth of tamoxifen resistant breast cancer. This new approach represents a potential therapeutic strategy for intrinsic tamoxifen-resistant patients and relapsed ERα-positive breast cancer patients. Our findings shed new light on the molecular mechanism of ATRA in overcoming tamoxifen resistance and warrant future preclinical and clinical studies of ATRA in treating the tamoxifen resistant breast cancers.

## Data Availability Statement

All datasets generated for this study are included in the article/Supplementary Material.

## Ethics Statement

The studies involving human participants were reviewed and approved by the Internal review and ethics boards of Sun Yat-sen Memorial Hospital. The patients/participants provided their written informed consent to participate in this study. The animal study was reviewed and approved by the Animal Care and Use Committee of Sun Yat-sen University.

## Author Contributions

M-LL and SH: conception and design. YL and YZ: development of methodology. YC, Z-ML, N-NL, and DL: acquisition of data. YC and SH: analysis and interpretation of data. M-LL and YY: writing, review, and/or revision of the manuscript. XZ and KL: technical and material support. M-LL and YY: study supervision.

## Conflict of Interest

The authors declare that the research was conducted in the absence of any commercial or financial relationships that could be construed as a potential conflict of interest.
