# Supplementary material for: Targeting Pin1 by All-Trans Retinoic Acid (ATRA) Overcomes Tamoxifen Resistance in Breast Cancer via Multifactorial Mechanisms
Source: Front Cell Dev Biol. 2019 Dec 6;7:322. doi: 10.3389/fcell.2019.00322 (PMC6908472; doi:10.3389/fcell.2019.00322)
Supplement: Supplementary file 1 [file Image_1.pdf]

Supplementary Material

Supplementary Figure 1

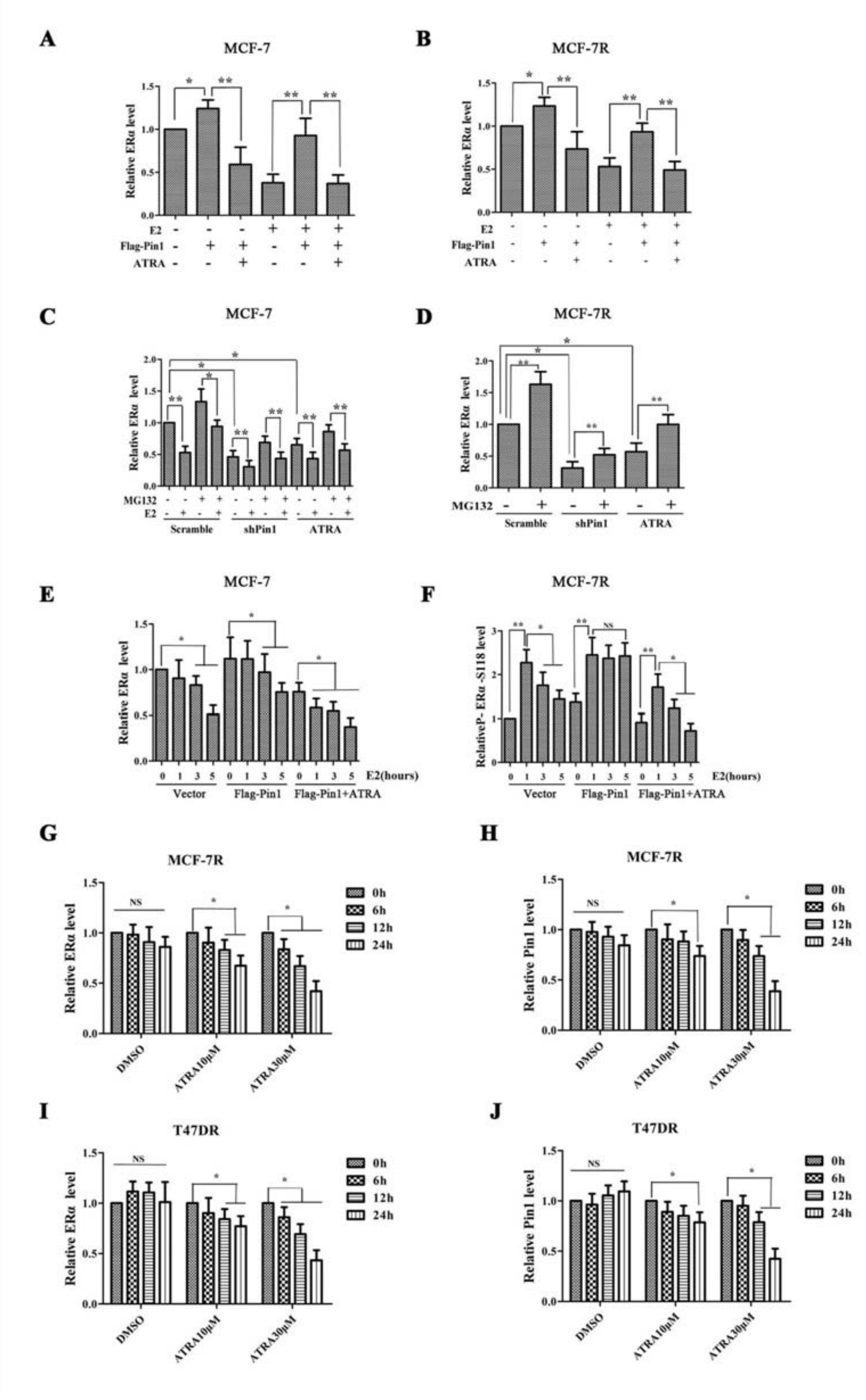

Supplementary Figure S1. ATRA promotes proteasome-mediated degradation of ERα by blocking Pin1, related to Figure 3.

(A, B) Quantification of ERα protein levels shows that ectopic expression of Pin1 upregulates ERα, but ATRA abrogates the effect. Western blot bands were quantified by densitometric scan and represented as a relative ratio to control samples. Data are represented as means ± SD for three independent experiments. $^*P < 0.05$, $^{**}P < 0.01$.

(C, D) Quantification of ERα levels shows that Pin1 knockdown or ATRA treatment promotes ERα degradation. $^*P < 0.05$, $^{**}P < 0.01$.

(E, F) Quantification of ERα and pS118- ERα levels shows that overexpression of Pin1 stabilizes pS118-ERα, but ATRA abrogates the effect. $^*P < 0.05$, $^{**}P < 0.01$.

(G-J) Quantification of ERα and Pin1 levels shows that ATRA promotes Pin1 and ERα degradation in MCF-7R and T47DR cells. $^*P < 0.05$.

Supplementary Figure 2

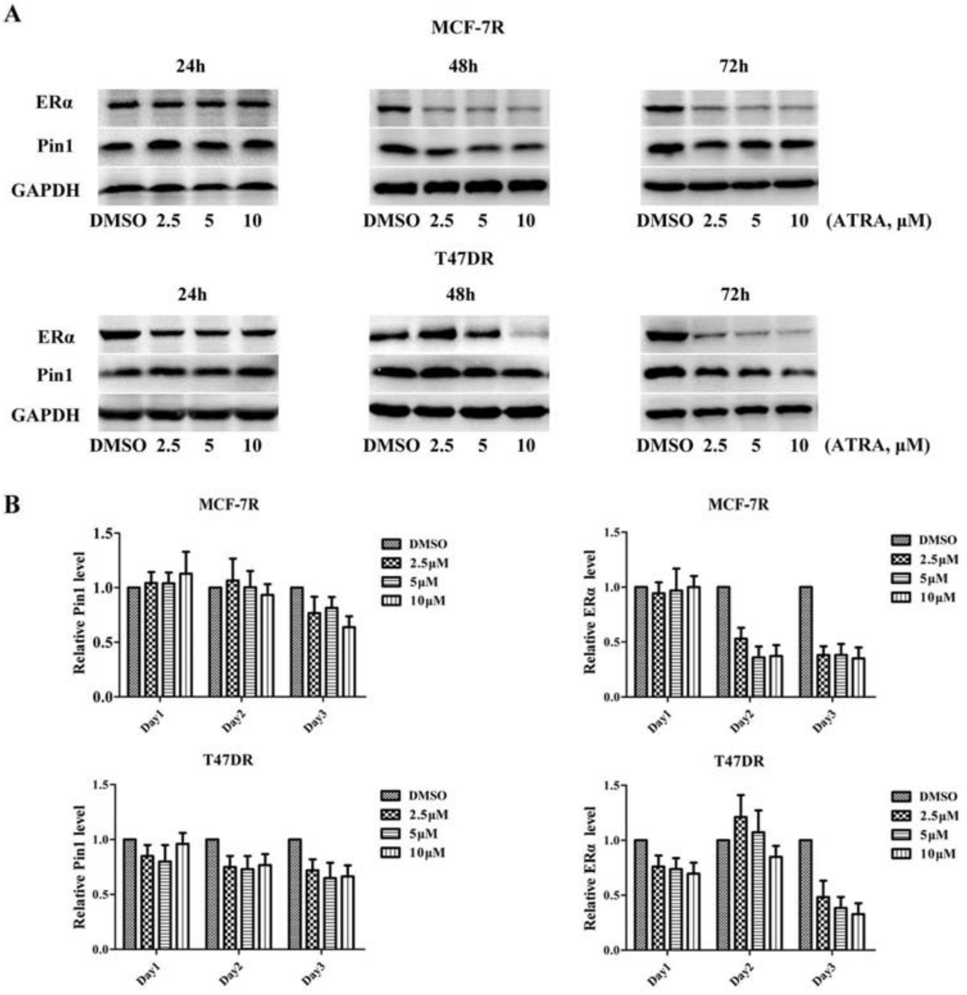

Supplementary Figure S2. ATRA treatment reduces Pin1 and ERα protein levels in tamoxifen resistant breast cancer cells.

(A) ATRA treatment reduces Pin1 and ERα protein levels. MCF-7R and T47DR cells were treated with increasing doses of ATRA for different length of time before harvesting.

(B) Densitometric value of western blot bands were quantified by Image J software. Data were represented as means ± SD for three independent experiments.

Supplementary Figure 3

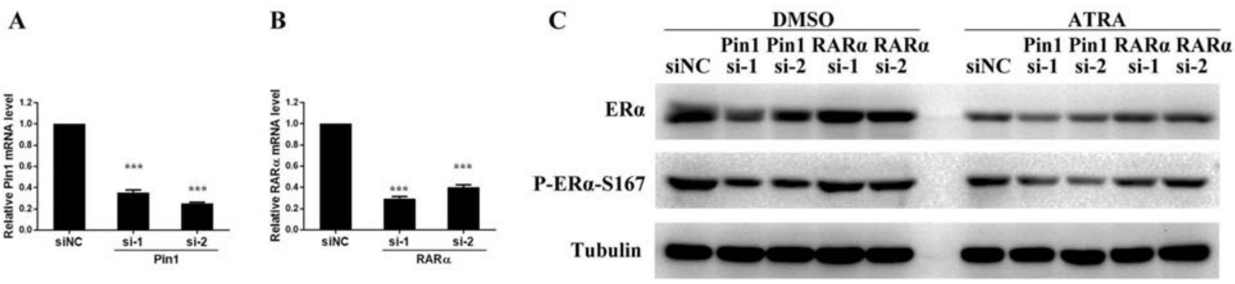

Supplementary Figure S3. Effects of Pin1 and RARα siRNAs on the level of ERα protein.

(A, B) Bar graphs shows the knockdown efficiency of siRNAs, as detected by qRT-PCR. ****P* < 0.001.

(C) The siRNAs of Pin1, but not RARα reduced the total and phosphorylated levels of ERα in MCF-7R cells.

Supplementary Figure 4

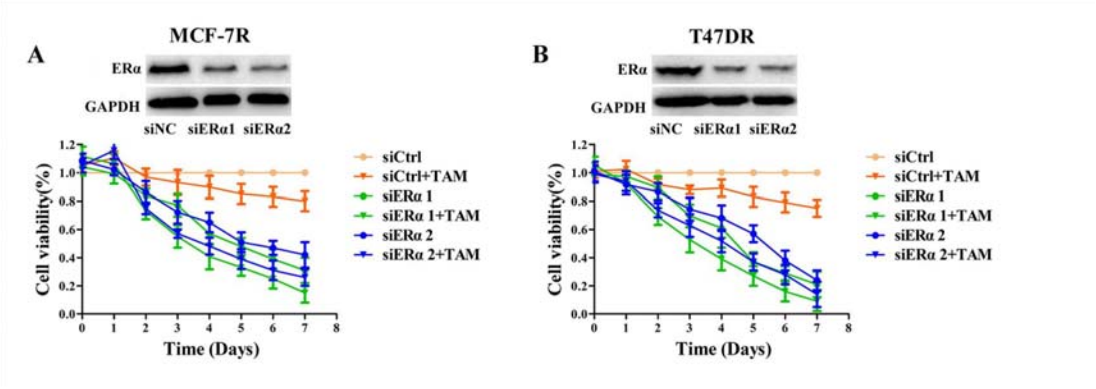

Supplementary Figure S4. Knocking down ERα inhibits the proliferation of TAMR cells, related to Figure 6.

(A, B) ERα siRNAs suppresses the proliferation of TAMR cells upon tamoxifen treatment. ERα was knocked down by two siRNAs in MCF-7R and T47DR cells.

Supplementary Figure 5

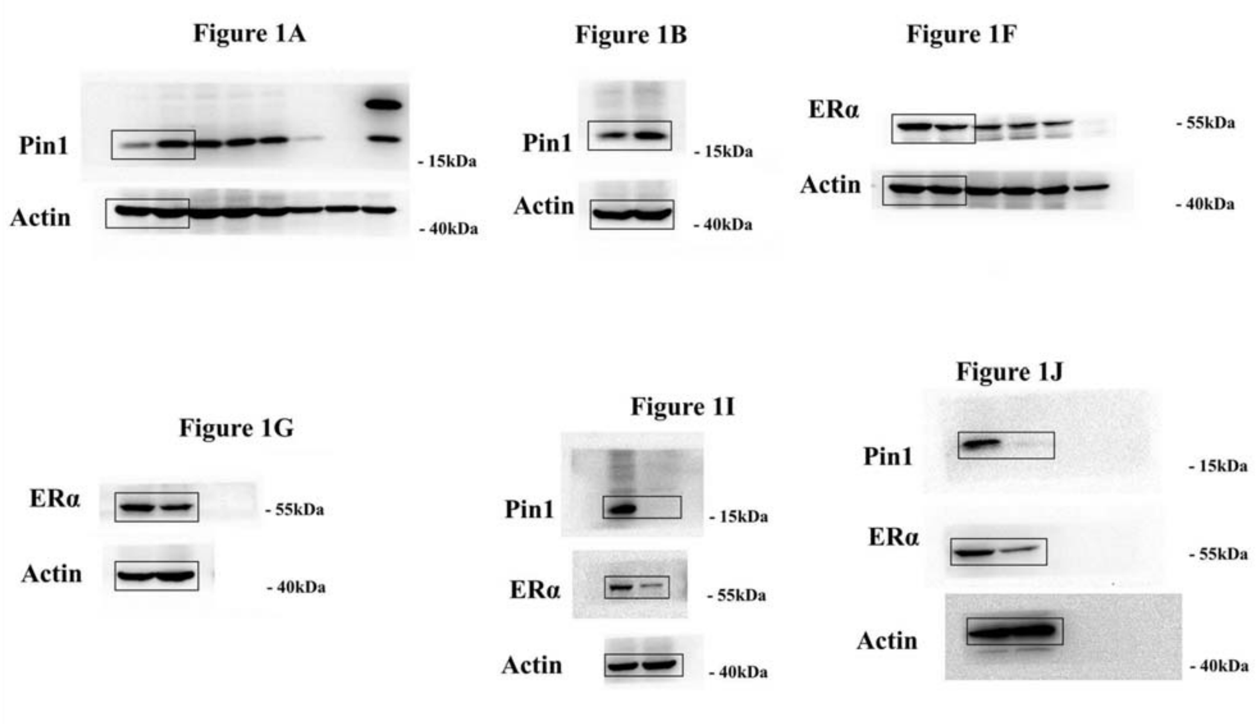

Full images of the blots in Figure 1. Images were obtained using BIO-RAD ChemiDoc XRS+ which directly scanned membranes developed with ECL reagents.

Supplementary Figure 6

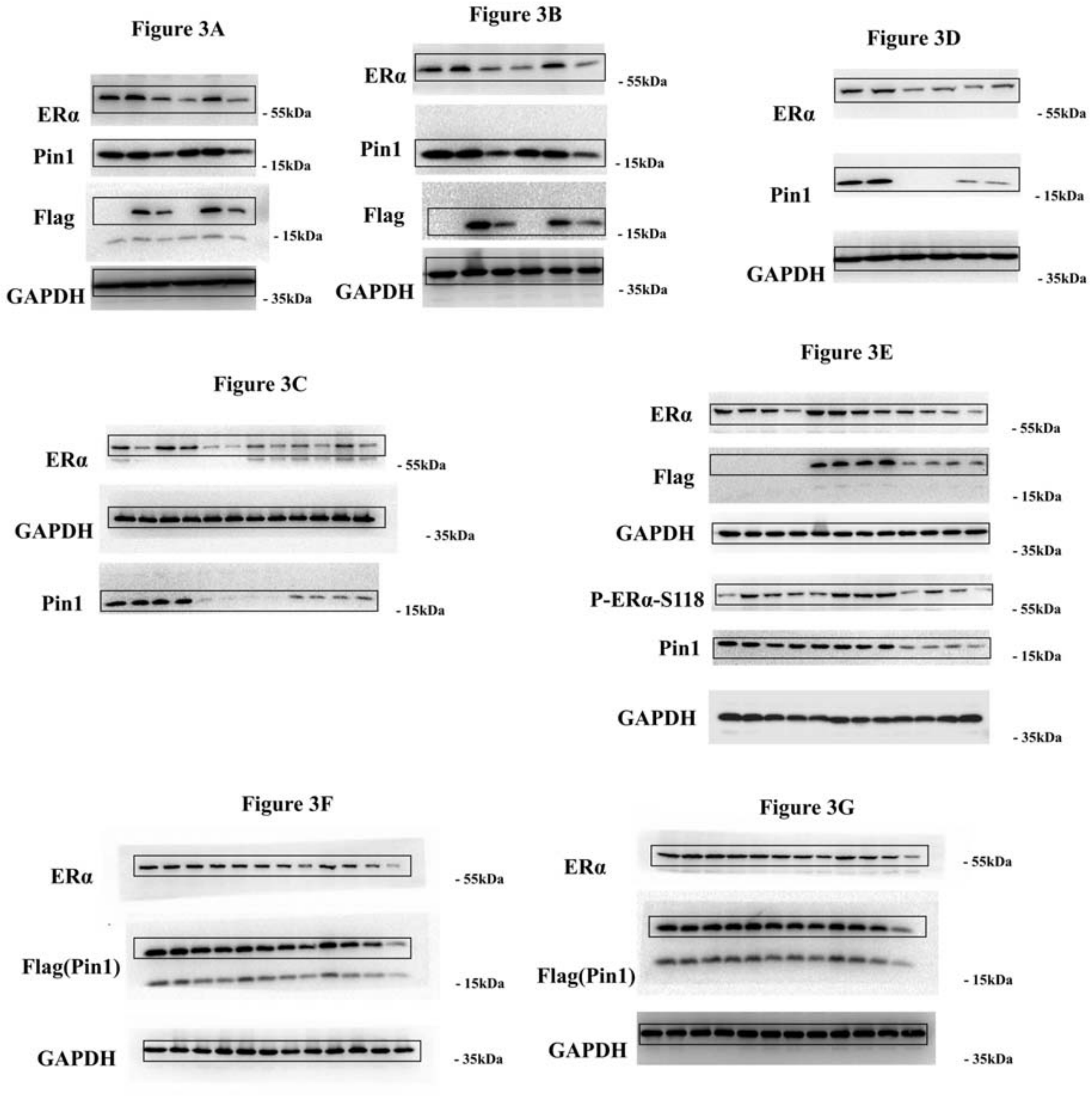

Full images of the blots in Figure 3. Images were obtained using BIO-RAD ChemiDoc XRS+ which directly scanned membranes developed with ECL reagents. The control GAPDH images were re-used in Figure 3E, with the original controls being shown here.

Supplementary Figure 7

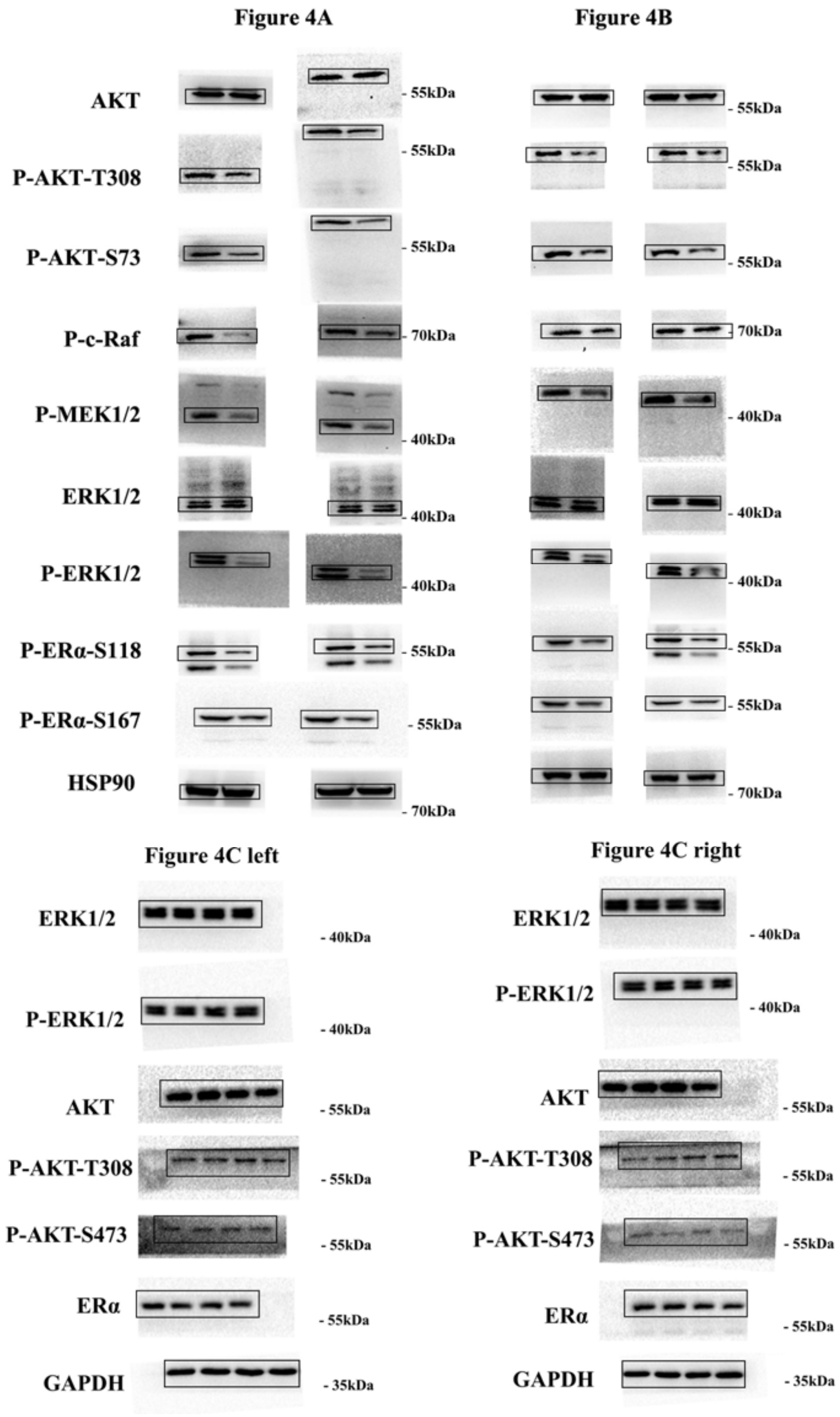

Full images of the blots in Figure 4. Images were obtained using BIO-RAD ChemiDoc XRS+ which directly scanned membranes developed with ECL reagents.

Supplementary Figure 8

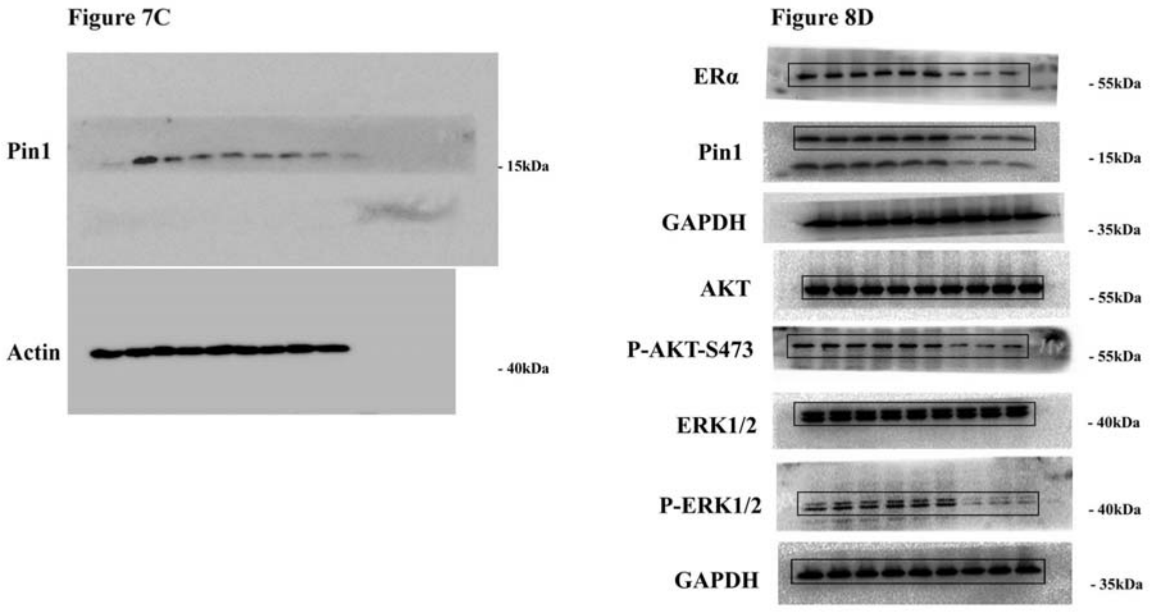

Full images of the blots in Figure 7 and 8. Images were obtained using BIO-RAD ChemiDoc XRS+ which directly scanned membranes developed with ECL reagents. The control GAPDH image was re-used in Figure 8, with the original control being shown here.

Supplementary Figure 9

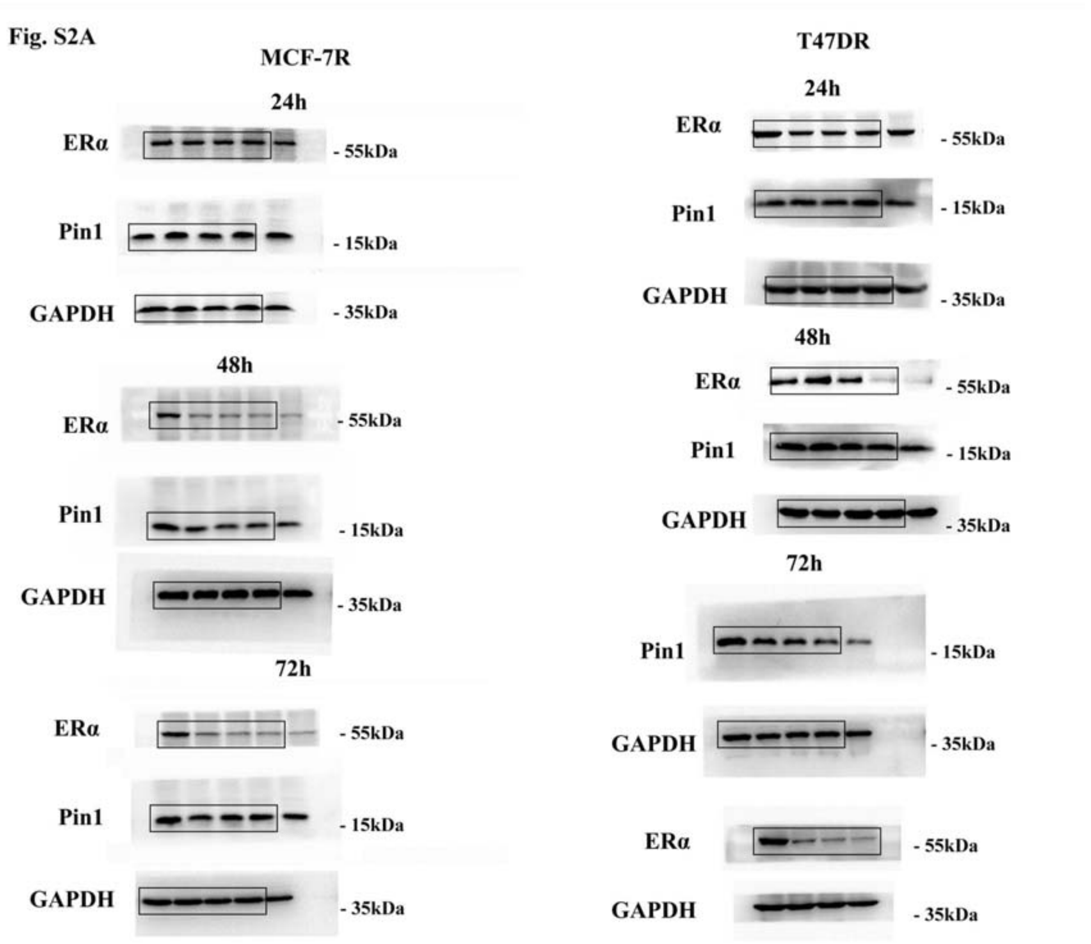

Full images of the blots in Figure S2. Images were obtained using BIO-RAD ChemiDoc XRS+ which directly scanned membranes developed with ECL reagents. The control GAPDH image was re-used in Figure S2A, with the original controls being shown here.

Supplementary Figure 10

Fig. S3C

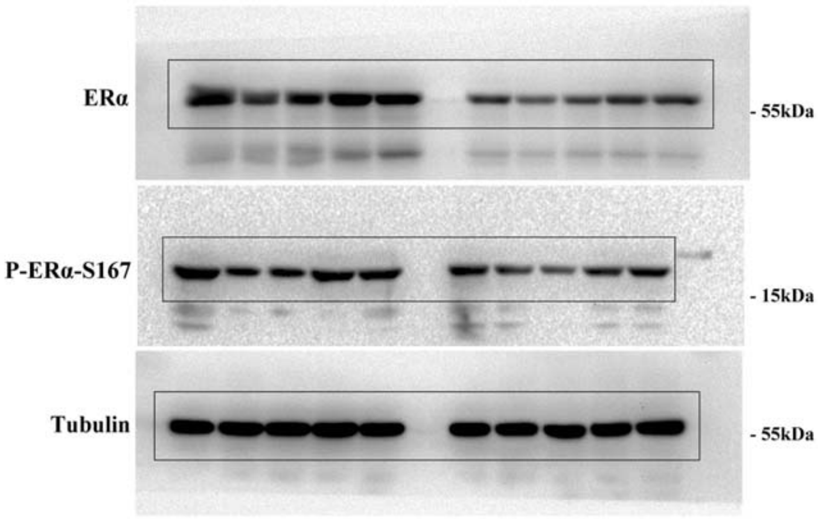

Fig. S4A

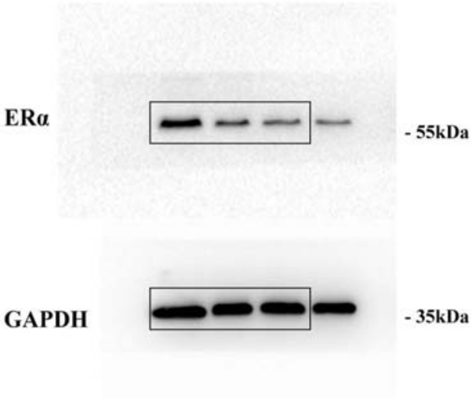

Fig. S4B

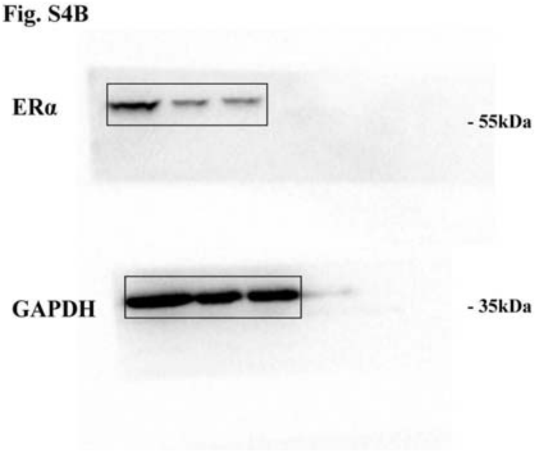

Full images of the blots in Figure S3 and S4. Images were obtained using BIO-RAD ChemiDoc XRS+ which directly scanned membranes developed with ECL reagents.
